# Characterization of street food consumption in palermo: possible effects on health

**DOI:** 10.1186/1475-2891-10-119

**Published:** 2011-10-28

**Authors:** Silvio Buscemi, Annamaria Barile, Vincenza Maniaci, John A Batsis, Alessandro Mattina, Salvatore Verga

**Affiliations:** 1 Department of Medicina Interna e Specialistica (DIMIS), Faculty of Medicine University of Palermo (Italy) - Policlinico "P.Giaccone", via del vespro, 129 - I-90127 Palermo, Italy; 2 Dartmouth College, Hanover, New Hampshire, USA; 3 Section of General Internal Medicine, Dartmouth-Hitchcock Medical Center, Lebanon, New Hampshire, USA

## Abstract

**Background:**

Street Food (SF) consists of out-of-home food consumption and has old, historical roots with complex social-economic and cultural implications. Despite the emergence of modern fast food, traditional SF persists worldwide, but the relationship of SF consumption with overall health, well-being, and obesity is unknown.

**Methods:**

This is an observational, cross-sectional study. The study was performed in Palermo, the largest town of Sicily, Italy. Two groups were identified: consumers of SF (n = 687) and conventional restaurant food (RES) consumers (n = 315). Study subjects answered a questionnaire concerning their health conditions, nutritional preferences, frequency of consumption of SF and a score relative to SF consumption ranging from 0 to 20 was calculated.

**Results:**

Body mass index (BMI, kg/m^2^) was significantly and independently correlated with the score of street food consumption (r = 0,103; p < 0.002). The prevalence of different diseases, including hypertension and type 2 diabetes, and the use of medications did not differ between the two groups. Milza (a sandwich stuffed with thin slice of bovine spleen and lung) consumers had a significantly higher prevalence of hypertension (12.2% vs 6.2% in non consumers; p < 0.005) and in this subgroup the use of anti-hypertensive drugs was inversely correlated with the frequency of milza consumption (r = 0.11; P = 0.010).

**Conclusions:**

This study suggests that SF consumption in Palermo is associated with a higher BMI and higher prevalence of hypertension in milza consumers. Further studies should evaluate whether frequent SF consumers have unfavourable metabolic and cardiovascular profile.

## Introduction

The increasing prevalence of obesity [1] and other cardiovascular risk factors [2] have been partially attributed to changing nutritional habits and reduced level of regular physical activity. The obesity epidemic is also influenced by social network phenomena characterized by behavioural person-to-person interactions [3]. Fast food consumption, defined as inexpensive food, sold by means of self-service systems or carry-out eating locales without waiter service [4], has been implicated as one potential reason for this epidemic [5,6]. It is energy dense, poor in nutritional and fibre content, high in glycemic load and often associated with large portion sizes [4,7].

Closely linked to fast food is another version of out-of-home food consumption termed *Street Food *(SF). This entity has old, historical roots with complex social-economic and cultural implications [8]. Street food is present even in less developed countries, and has occasionally been considered to be the hallmark of the early development of fast food [9,10]. It is quickly available and consumed, and generally affordable to large parts of the population.

Despite the emergence of modern fast food, traditional street food persists worldwide, especially in Europe and in Mediterranean countries [Maniaci V. Il cibo di strada e la comunità palermitana: indagine circa gli aspetti nutrizionali. University of Palermo, degree thesis for dietician, 2009]. In a broad sense, street food has often been described as having some elements of the Mediterranean Diet [11,12]. Few studies exist that consider the health and psychological effects of street food consumption in habitual consumers [13-26]. To our knowledge, no study has ever considered the relationship of street food consumption in the western world with overall health, well-being, and obesity. We undertook this study to characterize this relationship.

## Methods

This observational, cross-sectional study was performed in Palermo, the largest town of Sicily, Italy, with a population of 663.173 inhabitants. We identified two groups: consumers of SF and a control group consisting of conventional restaurant food consumers (RES). Subjects of the SF group were recruited among customers of a historical district of Palermo where SF is traditionally served (Antica Focacceria San Francesco, Palermo). RES subjects were recruited concurrently in the same time period, day of week, and hour of day as the SF group, in four restaurants quoted in specialized guides and present in the center of Palermo, where it is possible to consume complete meals according to a conventional sit-down dinner menu based on usual Mediterranean foods. These restaurants were contacted by the Confcommercio-FIPE, the representative organization of commercial activities. All restaurants permitted the study staff to approach and survey their customers.

From 17th September until 19th October 2009, every Thursday through Sunday, groups composed of physicians and dieticians asked study subjects to answer a questionnaire concerning their health conditions, general nutritional/dietary preferences/habits, usual frequency of consumption of SF or of foods offered on the restaurant menu in the past year. When requested, interviewers assisted customers in completing the questionnaire (< 10% of cases) when subjects requested information about diseases or medication consumption. In no case did the operators complete the questionnaires for the subjects. The four page questionnaire was administered in the Italian language, and was intended to be completed between 2-4 minutes. Inclusion criteria were subjects aged ≥ 18, born and resident in Palermo. We therefore excluded those participants < 18 years old, those who were not born in Palermo or non-residents of Palermo. There was no incentive provided to the subjects.

Height was listed on their national identify card when available (59%) or self-reported (41%). Self-reported body weight was listed on their registration card or directly obtained if requested by the subjects (22%) by means of a portable weight balance (SECA, cursa 818; Hamburg, Germany); body mass index (BMI) was calculated as body weight (kg)/height^2 ^(m).

An approved informed consent form was signed by each subject. The study protocol was approved by the Ethics Committee of the University Hospital Policlinico P. Giaccone of Palermo, Italy, and by the Committee for the Protection of Human Subjects at Dartmouth College, USA.

### Description of Street Food

The consumption of street food specialties unique to Palermo's tradition were considered [Maniaci V. Il cibo di strada e la comunità palermitana: indagine circa gli aspetti nutrizionali. University of Palermo, degree thesis for dietician; 2009] as briefly described in Table 1. A more complete gastronomic and nutritional description of street food specialties of Palermo's is available at the web address http://www.scribd.com/doc/60786261/Palermo-Street-Food. Subjects were asked if they consumed each of these foods (yes/not); self-reported frequency of consumption of each food was categorized less than or greater than once a month. Each of the 10 considered foods was scored 0 = never consumed, 1 = once a month or less, 2 = more than once a month; the sum of single scores ranged therefore from 0 and 20.

**Table 1:** Description of street food specialties of Palermo's tradition

Food	Description
**Panelle**	pancakes of chickpeas flour
**Rascatura**	obtained from the residual panelle's dough left in the edges of the patty used to cook them
**Crocchè**	fried cylinders of mashed potatoes
**Arancine**	fried rice spheres stuffed with sauce and meat or mozzarella cheese and ham
**Focaccia with milza**	a sandwich stuffed with thin slice of bovine spleen and lung, ricotta cheese and slivers of caciocavallo, very savoury oblong-shaped cheese of Southern Italy
**Sfincione**	a soft leavened dough seasoned with tomato, anchovies, onion, olive oil and oregano
**Frittola**	consists of small pieces of fat, cartilages and bit of meat detached by the discards of slaughter that, first of all, are fried and then boiled
**Musso and Quarume**	the feet, the jaw, the breast and other parts of the calf cut into bits and boiled, they constitute the *musso*; the same ingredients of the musso can be served with vegetable broth and in this way they are called the *quarume*.
**Stigghiole**	guts of calf twisted into strips of fat and onion, pierced in a spit and roasted

### Co-Morbid conditions

All subjects answered questions relative to regular medication consumption and concomitant diseases specifically concerning the presence of the following: hypertension, type 1 and type 2 diabetes mellitus, dyslipidemia, coronary heart disease, stroke, gastro-intestinal diseases including dyspepsia, gastric or duodenal ulcers, hiatal hernia, inflammatory bowel disease, gallstones, kidney stones, chronic renal failure, chronic respiratory diseases,. Subjects were also asked whether they had followed a low calorie diet, and if so, whether it was prescribed by a health professional or was self-initiated. Finally, subjects were asked whether they ever used weight-loss medications. Medications were self-reported with their trade name and a physician coded each drug in its respective category.

### Statistic analysis

All data are expressed as mean ± sd or as prevalence (%). Variables relative to single food consumption, diseases and drug categories were dichotomized (1 = yes; 2 = no). Continuous variables were assessed using Student's unpaired *t*-test and categorical variables using Pearson's χ2 test. Linear regression analysis assessed the relationships between variables; the independency of associations between variables was assessed using multiple regression analysis. A two-tailed p < 0.05 was considered statistically significant. Normal distribution of continuous variables was tested by the Kolmogorov-Smirnow test. All analyses were performed using SYSTAT (Windows version 11.0; Systat Software Inc., San Jose, CA, USA).

## Results

**Figure 1:**
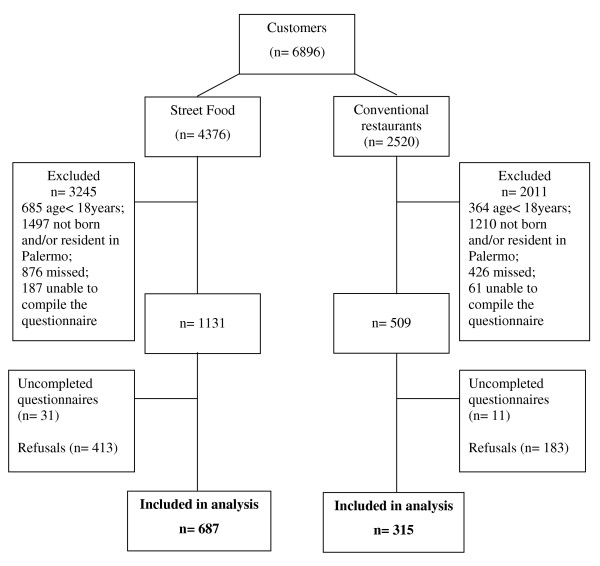
**Subjects selection flow among the customers of conventional restaurants or of a street food place**.

Of a total of 1,002 subjects with complete data, the SF group consisted of 687 subjects and RES group 315 subjects (Figure 1). Age, height, weight and BMI were normally distributed.

### Demographics

Demographic characteristics are reported in Table 2. The group's marital status differed and the number of offspring was significantly higher in the SF group. The SF group had a higher prevalence of unemployed and employed subjects and of housewives.

**Table 2:** Demographic characteristics of the study groups^1^.

	**Groups**		
	**Street Food**	**Restaurant**	**P**^**2**^
		
males/females (n)	321/366	141/174	0.61
age (years)	37.6 ± 13.8	37.9 ± 14.1	0.75
body weight (kg)	69.9 ± 14.8	68.5 ± 15.8	0.17
BMI (kg/m^2^)	24.3 ± 4.1	23.7 ± 4.4	< 0.05
marital status (%):			< 0.005
single	44.4	47.3	
married	51.1	47.4	
divorced	2.9	3.8	
widow/er	1.6	1.5	
offspring (n)	1.1 ± 1.3	0.8 ± 1.0	< 0.001
employment status (%):	n = 905		< 0.001
student	21.6	25.0	
unemployed	3.4	1.7	
housewife	13.4	6.0	
employed	42.7	37.5	
manager/professional	14.9	26.7	
retired	4.0	3.1	
smokers (%)	35.0	33.3	0.67
previous diet therapy (%):			0.053
self-prescribed	31.9	27.9	
prescribed	20.2	27.0	
previous or current use of weight-reduction medications (%)	6.1	4.4	0.36

^1 ^Mean ± sd or percentage. ^2 ^Student's unpaired *t*-test or Pearson's χ^2 ^test, as appropriate. BMI: body mass index.

### Food preferences

**Figure 2:**
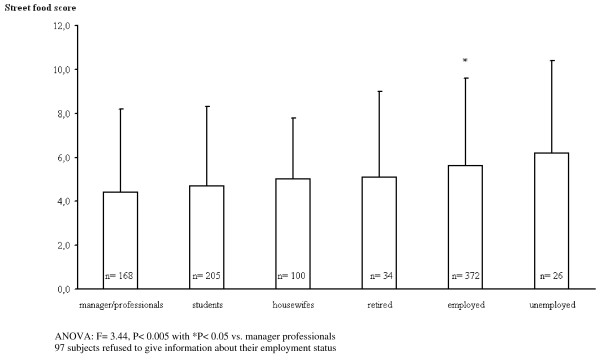
**Street food score and employment status**.

The score of street food consumption significantly (F = 3.44; p < 0.005) changed according to the employment status (Figure 2). The prevalence of subjects who reported following at least one low calorie diet in their lifetime was no different between groups (p = 0.053), despite a higher percentage of subjects in the SF who had followed a self-prescribed diet. The SF score was 5.1 ± 3.8 with a median value of 5 (range 0-20); interquartile ranges were, respectively, 1^st ^quartile: 0-1; 2^nd ^quartile: 2-4; 3^rd ^quartile: 5-6, 4^th ^quartile: 7-20. SF consumption score was higher in the SF group (6.6 ± 3.5 [median 6.0; range of 1-20] vs 1.8 ± 2.2 [median 1.0; range of 0-10]; P < 0.001), yet overall, both groups consumed each street food with the same order of preference (Additional file 1 Table A). Beer (46.4 vs. 8.9%; p < 0.001) and soft drinks (31.9 vs. 11.4%; p < 0.001) were the preferred beverages in SF subjects, while water (14.4 vs 36.8%; p < 0.001) and wine (7.3 vs. 42.9%; p < 0.001) were consumed more often in RES subjects. No significant correlation was observed between the score of SF consumption and any of the diseases considered in the study. Age was inversely and independently correlated with the score of street food consumption (r = -0.069; p < 0.05).

### Health and foods

**Figure 3:**
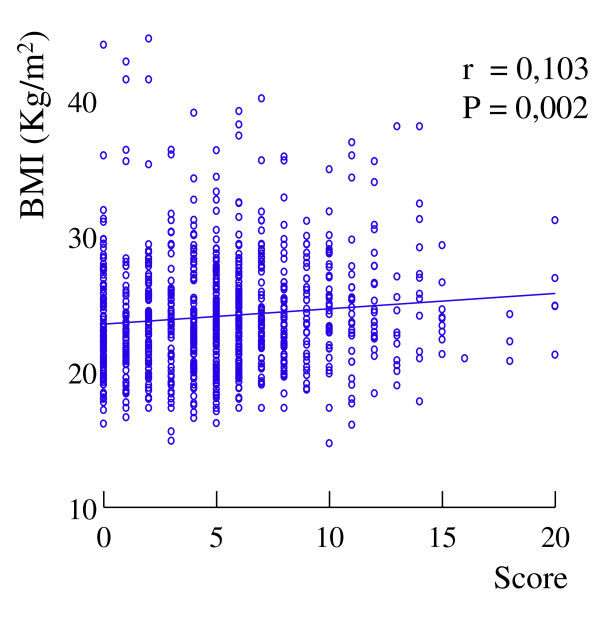
**Correlation between the score of street food consumption and body mass index (BMI)**.

The SF group had a significantly higher BMI than the RES group (Table 2). BMI was significantly and independently correlated with the score of street food consumption (Figure 3, Table 3), and independently associated with age, sex, and offspring number (Table 3) that gave an R^2^= 0.26. The correlation between BMI and the score of street food consumption was significant (r = 0.10; p < 0.05) also in addition to considering only the subjects of the SF group. In this latter group, the BMI increased significantly across the tertiles of street food score (23.8 ± 4.3 vs 23.9 ± 4.0 vs 24.7 ± 4.1 kg/m^2^; F = 3.42; p < 0.05), with third tertile significantly different (p < 0.05) from first and second tertiles respectively. The SF score was independently correlated with age, sex, and body weight (Table 4). The presence of at least one disease was independently associated with age, sex and BMI (Table 3) that gave an R^2^= 0.11.

**Table 3:** Independent predictors by multiple regression analysis of BMI, presence of at least one of the considered diseases and hypertension.

Variables			
**Outcome**	**Independent variable**	**coefficient β**	**P**

**BMI**			
	constant	25.037	< 0.001
	age (years)	0.071	< 0.001
	sex (1 = m; 2 = f)	-2.862	< 0.001
	street food (score)	0.064	< 0.05
	offspring (n)	0.503	< 0.001
**Diseases** **(1 = yes; 2 = not)**			
	constant	2.415	< 0.001
	age (years)	-0.010	< 0.001
	sex (1 = m; 2 = f)	-0.085	< 0.01
	BMI	-0.010	< 0.01
	street food (score)	-0.001	0.715
**Hypertension** **(1 = yes; 2 = not)**			
	constant	2.169	< 0.001
	age (years)	-0.008	< 0.001
	sex (1 = m; 2 = f)	0.028	0.127
	street food (score)	-0.001	0.632

BMI: body mass index; m = male; f = female.

Diseases: at least one of the following: hypertension, type 1 and type 2 diabetes mellitus, dyslipidemia, coronary heart disease, stroke, gastro-intestinal diseases including dyspepsia, gastric or duodenal ulcers, hiatal hernia, inflammatory bowel disease, gallstones, kidney stones, chronic renal failure, chronic respiratory diseases

Variables coded as 2 are the reference category.

**Table 4:** Independent Predictors of the street food score by linear regression model

Variables	coefficient β	P
constant	6.1	< 0.001
age (years)	-0.03	0.004
sex (1 = m; 2 = f)	-0.99	0.002
body weight (kg)	0.02	0.016

m = male; f = female

Hypertension was independently associated with age (R^2^= 0.15; Table 3). Milza consumers (n = 599) had a significantly higher prevalence of hypertension than non consumers (n = 405) (12.2 vs 6.2%; p < 0.005) and in this subgroup the use of anti-hypertensive drugs was inversely correlated with the frequency of milza consumption (r = 0.11; p < 0.01). Disease prevalence and medication usage were comparable between the two groups (Additional file 1 Table B and C). By combining all subjects into two groups according to the median value of SF score or by using tertiles of SF score, no significant difference was observed in any disease rate (data not shown).

## Discussion

Our findings suggest that subjects who consume Street food are associated with higher prevalence rates of BMI and hypertension than the restaurant eating group. We observed a relationship between SF consumption and BMI supporting the hypothesis that SF may be a proxy indicator of other components of an unhealthy lifestyle as behaviours have been documented to aggregate [27]. These results parallel those previously reported for fast food [4,6] suggesting that frequent consumption of street food is equally an unhealthy behaviour.

Despite significant, the influence of SF consumption was rather limited. We demonstrated that body weight was a highly significant predictor of elevated SF score. We additionally examined whether SF score predicted other outcomes including body mass index. These results may indicate that a diet that includes SF consumption may lead to reduction of other possible healthier foods. Additionally, these results provide preliminary information that the relationship with weight/obesity may in fact be bi-directional. Both the number of SF specialities consumed and the frequency of their consumption may influence the SF score. Subjects in the highest quartile of SF score reported values ≥ 7. From the regression analysis (Table 4), approximately 100 kg of body weight were associated with 2 points of SF score, corresponding to the habitual consumption of two specialties of SF once a month or less or, alternatively, to the consumption of one specialty of SF more than once a month. Therefore, the SF score probably reflects a tendency towards a nutritional style rather than the amount of habitual energy intake. However, further validation studies would be needed to better examine this relationship.

The relationships between SF consumption, BMI and health status may have been influenced by a selection bias that is hardly avoidable in similar studies. Subjects frequenting the SF place and restaurants respectively, may differ, mainly on socioeconomic characteristics, yet specific data other than employment was not canvassed. Observed differences (Table 2) concerning employment, marital status and offspring number may also reflect such differences in outcomes independently from dietary factors. The SF score changed according to employment status so thus manager and professionals had a significantly lower consumption of street food than the employed (Figure 2). Unfortunately, we were unable to further parse the data in this group to further explore this association. The significant positive correlation between the SF score and BMI even within the SF group leads us to believe sampling bias may not be socioeconomic related suggesting that diet style influenced body size probably independently from socioeconomic status.

Our study describes the consumption of local traditional SF on obesity and medical illnesses and is unique in that it addresses a critical knowledge gap. Our study, to our knowledge, is the first to describe such patterns in a European city. Such information is currently only available for modern fast food consumption [4,28]. Our results further the understanding that SF has possibly contributed to the sustainability of low-income people and is analogous to modern fast food for the effects on some aspects concerning health. Furthermore, these results may be useful in targeting future specific interventions in our local geographic area.

The few published studies on SF have exclusively considered only the description of food, chemical or microbiological contaminants [13-18], its limited nutritional value [19,20,29] or have been described anecdotally [21,22]. In general, street food consumption is perceived as an unhealthy behaviour [23] which exerts a strong appeal [24] and may be advisable to limit its consumption. It has been also described that is in some cases, subjects underwent psychological conditioning strategies for decreasing SF consumption [25,26].

Despite the wide availability of SF in the study region, we recruited the SF group in the oldest place of SF consumption of Palermo which now functions as a SF restaurant to obtain a more homogeneous and comparable group with respect to the group RES. The manner in identifying subjects reduces, to one extent, the possible psycho-social-cultural confounding factors (for instance alcohol abuse or very low income). The frequency of different street food consumption was similar in both groups, suggesting that the Palermo's street food consumption is a diffuse cross-cultural and socio-cultural phenomenon. Our results also suggest that as one ages, SF consumption is reduced as has previously been demonstrated whereby eating habits become healthier with aging [30,31].

This study has some important limitations. Our data is self-reported and subject to recall and measurement bias. While we relied on these self-reported measures, undoubtedly this introduces bias as to the accuracy of the values/medications reported. Structured interviews and assessments would be required, which would not be practical in this study protocol. However, the manner in which we ascertained the data was similar in both groups and emerging data does suggest that self-reported BMI may be accurate [32]. Trust in the health-care system is known to be related to self-rated health [33,34]. Recent investigations demonstrate that Italians hold trust in their national health care system [35] and we are hopeful that less reporting bias concerning self-rated health may have occurred in this study. The manner in recruiting the two groups directly in the place of food consumption did not allow further clinical data gathering; we were also limited in time by needing to ask our questionnaire in only a few minutes. Studies have demonstrated that short surveys often have higher completion rates [36]. We acknowledge that the demographic characteristics of each group differed, which may account for participant's response to questions and may be reflective in their food consumption.

Other factors, including time of the year may influence the extent of SF consumption. Nonetheless, the comparability of anthropometric and demographic characteristics and its relationship to SF consumption is unique to our own study. Finally, the calculated SF score was not validated but still provides interesting information. The SF score has other intrinsic limitations, for instance someone who eats street food most days of the week but only eats one item would only have a score of 2, however we may consider this as an extreme situation that is expected to have no high influence on the results given the relatively high number of subjects included in this study. We acknowledge that further validation studies would be needed to allow an accurate interpretation of what an elevated SF score means.

Due to our sampling strategy, the study cannot be generalized and our results must be referred only to local people whose nutritional habits include different degree of street food consumption. Whether our results parallel similar food practices in other countries is unknown but may be a subject for further investigation.

The prevalence of hypertension and diabetes (Additional file 1 Table B) was lower than that in the general local population [37,38]. Studies have shown that those with diagnosed hypertension and diabetes tend to have better eating habits than those with undiagnosed disease [39-41]. The study subjects were younger than in the general population who have been documented to have poor eating habits [42]. Secondly, our data is self-reported and subject to recall and measurement bias.

Although we describe associations of specific foods with given medical conditions, our data needs to be confirmed with longitudinal studies. The higher prevalence of hypertension in subjects eating focaccia with milza may be linked with the fact that this food is cooked with lard, and is characterized with high contents of saturated fatty acids. These entities are associated with oxidative stress, endothelial dysfunction and cardiovascular risk [43,44]. On the contrary, the main ingredient of panelle is chick-pea floor that is rich in polyunsaturated fatty acids, that of arancine is rice and potatoes are the main ingredient of crocchè. Yet without knowing the exact nutritional content of each of the foods concerned in our study, our statements are purely speculative.

## Conclusions

SF consumption may be associated with elevated BMI and prevalence of hypertension and appears to be a diverse nutritional habit in all socioeconomic strata. This study outlines a possible approach for defining and recognizing specific social-environmental aspects of population nutritional culture that are now recognized to have an important role in supporting health-promoting patterns of behaviour [45]. Our results are not meant to be definitive in nature, but exploratory due to sample size limitations. Further studies should evaluate whether frequent street food consumers have unfavourable metabolic and cardiovascular profile.

## List of abbreviations

BMI: body mass index; RES: conventional restaurant food; SF: street food.

## Competing interests

The authors declare that they have no competing interests.

## Authors' contributions

SB was the main author of the manuscript and contributed to the design of the study, preparation of protocols, statistical analyses, interpretation of data and preparation of the manuscript. AB contributed to preparation of protocols, data collection and interpretation of data. VM contributed to preparation of protocols, data collection and interpretation of data. JAB contributed to the interpretation of data and preparation of the manuscript. AM contributed to data collection and preparation of the manuscript. SV contributed to interpretation of data and preparation of the manuscript. All authors read and approved the final manuscript.

## Supplementary Material

Additional file 1 **Tables A, B and C**. The file contains data concerning the street food preferences, the prevalence of reported diseases and the use of medications in the two cohorts of Street Food consumers and of Restaurant Food consumers. Click here for file
