# Supplementary material for: Characterization of street food consumption in palermo: possible effects on health
Source: Nutr J. 2011 Oct 28;10:119. doi: 10.1186/1475-2891-10-119 (PMC3228696; doi:10.1186/1475-2891-10-119)
Supplement: Additional file 1 — Tables A, B and C. The file contains data concerning the street food preferences, the prevalence of reported diseases and the use of medications in the two cohorts of Street Food consumers and of Restaurant Food consumers. [file 1475-2891-10-119-S1.DOC]

**The appendix**

| Table A. Street food preferences in the two cohorts of Street Food (SF) consumers and of restaurant food consumers (RES). (percentages). | | | |
| --- | --- | --- | --- |
|  | **SF** | **RES** | P^2^ |
| Panelle | 87.2 | 43.5 | < 0.001 |
| Arancine | 83.6 | 37.5 | < 0.001 |
| Crocchè | 77.9 | 27.3 | < 0.001 |
| Focaccia with milza | 76.9 | 22.5 | < 0.001 |
| Sfincione | 75.7 | 24.8 | < 0.001 |
| Stigghiole | 46.3 | 11.7 | < 0.001 |
| Musso | 28.5 | 5.1 | < 0.001 |
| Caldume | 23.3 | 4.1 | < 0.001 |
| Frittola | 15.3 | 2.2 | < 0.001 |
| Rascatura | 10.3 | 3.2 | < 0.001 |
| ^2^ Pearson’s χ^2^ test | | | |

| Table B. Prevalence of reported diseases in the two cohorts of Street Food (SF) consumers and of restaurant food consumers (RES). (percentages). | | |
| --- | --- | --- |
|  | **SF** | **RES** |
| diseases: |  |  |
| hypertension | 10.8 | 7.6 |
| coronary heart disease | 0.7 | 1.3 |
| Stroke | 0.3 | 0.3 |
| type 1 diabetes | 0.7 | 0.6 |
| type 2 diabetes | 1.5 | 1.9 |
| dislipidemia | 2.8 | 2.2 |
| gastro-intestinal diseases | 21.0 | 16.8 |
| gallstones | 0.7 | 2.2 |
| asthma | 4.1 | 1.9 |
| chronic renal failure | 0.0 | 0.3 |
| food allergies or intolerances | 6.4 | 6.7 |
| cancer | 0.9 | 1.0 |
| No significant difference at any point (Pearson’s χ^2^ test.) | | |

| Table C. Use of medications in the two cohorts of Street Food (SF) consumers and of restaurant food consumers (RES). (percentages). | | |
| --- | --- | --- |
|  | **SF** | **RES** |
| medications (%): |  |  |
| anti-hypertensives | 7.3 | 7.3 |
| ace-innibitors/sartanes | 5.8 | 6.0 |
| beta-blockers | 1.7 | 1.6 |
| alpha-blockers | 0.3 | 0.0 |
| diuretics | 1.7 | 1.0 |
| calcium-blockers | 1.6 | 1.0 |
| anti-platelets | 1.2 | 2.5 |
| oral anti-coagulants | 0.0 | 0.3 |
| oral hypoglycemic drugs | 0.9 | 1.6 |
| Insulin | 0.6 | 0.0 |
| statines | 0.1 | 0.0 |
| fibrates | 0.9 | 1.0 |
| omega-3 | 0.3 | 0.6 |
| anti-acids, H_2_ antagonists | 7.7 | 6.0 |
| No significant difference at any point (Pearson’s χ^2^ test.) | | |
